# Microglia in C9orf72–associated amyotrophic lateral sclerosis: More or less active?

**DOI:** 10.4103/NRR.NRR-D-25-01831

**Published:** 2026-01-02

**Authors:** Björn F. Vahsen, R. Jeroen Pasterkamp

**Affiliations:** Oxford Motor Neuron Disease Centre, Nuffield Department of Clinical Neurosciences, University of Oxford, John Radcliffe Hospital, Oxford, UK; Kavli Institute for Nanoscience Discovery, University of Oxford, Dorothy Crowfoot Hodgkin Building, Oxford, UK; Department of Translational Neuroscience, University Medical Center Utrecht Brain Center, Utrecht University, Utrecht, The Netherlands

Amyotrophic lateral sclerosis (ALS) is a neurodegenerative disease characterized by motor neuron (MN) loss and muscle wasting, ultimately leading to death due to respiratory failure. A hexanucleotide (GGGGCC) repeat expansion (HRE) in *C9orf72* is the most common genetic cause of ALS (C9-ALS) and frontotemporal dementia. *C9orf72* HRE causes ALS through different mechanisms, which include reduced C9orf72 expression, the generation of RNA foci and dipeptide repeat proteins (DPRs), and TAR DNA-binding protein 43 (TDP-43) pathology. A large body of experimental work supports a role for microglial changes in C9-ALS. For example, human post-mortem analyses show microglial tissue infiltration, *C9orf72* is strongly expressed in microglia, and loss of *C9orf72* leads to lysosomal accumulation and altered pro-inflammatory responses in mice. Furthermore, recent single-cell transcriptomic analysis of human C9-ALS microglia reports an impaired transition of microglia towards a reactive cell state, which is supported by observations in C9-ALS organoid-derived microglia (oMG).

**Figure 1:**
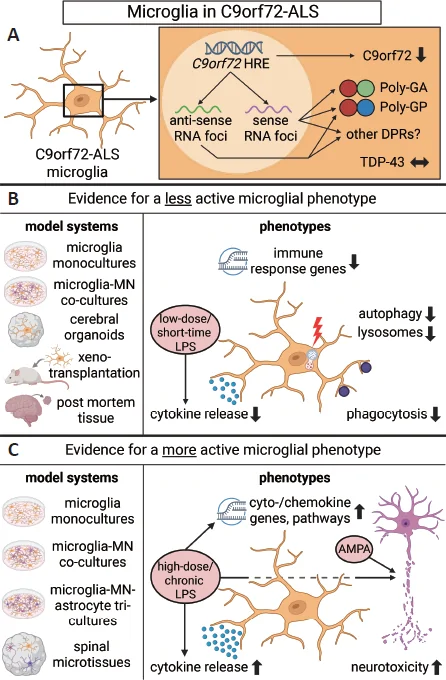
An overview of model systems and evidence demonstrating a more or less active microglial phenotype in *C9orf72*–associated ALS. (A) *C9orf72* hexanucleotide repeat expansion-associated loss-of-function (reduced C9orf72 expression) and gain-of-function hallmarks (sense and anti-sense RNA foci, dipeptide repeat proteins (DPRs) Poly-GA and Poly-GP) have been consistently found in *C9orf72*-ALS (C9-ALS) microglia. (B) A less active microglial profile in C9-ALS has been described in iPSC-derived microglia monocultures, co-cultures with motor neurons (MNs), cerebral organoids, xenotransplantation models, and post mortem tissue. Observed phenotypes include reduced expression of immune response genes, low cytokine release upon low-dose or short-term lipopolysaccharides (LPS) priming, reduced autophagy and lysosomes, and decreased phagocytosis. (C) iPSC-derived microglia monocultures, co-cultures with MNs, tricultures with MNs and astrocytes, and 3D spinal microtissues have provided evidence for a more active microglial phenotype in C9-ALS. These setups have demonstrated increased cytokine/chemokine pathways and higher cytokine release after high-dose and long-term stimulation with LPS, as well as microglial neurotoxicity towards MNs. Created with BioRender.com. ALS: Amyotrophic lateral sclerosis.

It has also become clear that mouse and human microglia are remarkably distinct. Further, microglia derived from (post-mortem) brain tissues may be more mature than those studied in culture, but at the same time provide different insights into the disease process (i.e., disease end-stage versus developmental stage). Therefore, several recent studies have used induced pluripotent stem cell (iPSC)-derived human microglia to study C9-ALS. This work has demonstrated gene expression changes, impaired phagocytosis, altered immune responses, defective initiation of autophagy, and a toxic microglial effect on spinal MNs (**Figure 1**). However, although these studies provide important insight into how *C9orf72* HRE affects microglia, apparent discrepancies exist. Here, we summarize and discuss recent insights into microglial dysfunction in C9-ALS with an emphasis on the most recent studies and those using human microglia. Further, we discuss discrepancies and provide directions for future research.

**Pathological hallmarks in C9-ALS microglia:** Important observations have been made in C9-ALS microglial monocultures. One key question that has been comprehensively addressed is whether the pathological hallmarks of the *C9orf72* HRE are present in microglia. C9orf72 is more highly expressed in human microglia than in neurons, both in iPSC-derived cells (Banerjee et al., 2023; Vahsen et al., 2023) and postmortem tissue (Masrori et al., 2025). There is consensus that *C9orf72* HRE decreases C9orf72 expression in microglia, at least at protein level (ranging from **~**30%–50%) (Banerjee et al., 2023; Lorenzini et al., 2023; Vahsen et al., 2023; Rostalski et al., 2024; Ljubikj et al., 2025; Gao et al., 2026; Mearelli et al., 2026). C9-ALS microglia further express sense- and anti-sense RNA foci, with higher abundance of the latter (Lorenzini et al., 2023; Vahsen et al., 2023; Rostalski et al., 2024; Ljubikj et al., 2025). A subset of DPRs (Poly-GA, -GP) has been detected in microglia, albeit at lower levels than in neurons (Lorenzini et al., 2023; Vahsen et al., 2023; Rostalski et al., 2024; Gao et al., 2026; Mearelli et al., 2026). A comprehensive analysis of all five DPR species within microglia remains to be conducted. There is thus far no evidence that *C9orf72* HRE triggers mislocalisation or intrinsic aggregation of TDP-43 within microglia (Lorenzini et al., 2023; Vahsen et al., 2023; Rostalski et al., 2024; Gao et al., 2026), suggesting that TDP-43 proteinopathy may be a primary neuronal hallmark of the disease. However, more subtle TDP-43 nuclear loss-associated splicing changes remain to be assessed in microglia. Together, these studies (with a variety of independent C9-ALS donors and differentiation protocols) are remarkably consistent and provide evidence that *C9orf72* HRE induces both loss- and gain-of-function effects in human microglia.

**Functional microglial defects:** To determine whether *C9orf72* HRE indeed alters microglial biology, experiments in monocultures have focused on classic microglial functions (phagocytosis, inflammatory profile, neurotoxicity) and pathways previously associated with known functions of C9orf72 in neurons (endolysosomal pathway, metabolism). Several studies have assessed phagocytosis, with varying results. Reduced phagocytosis was demonstrated by two studies using the same monoculture differentiation protocol (Banerjee et al., 2023; Ljubikj et al., 2025) and in microglia sorted from organoids (Ljubikj et al., 2025). In contrast, other studies report no difference (Lorenzini et al., 2023) or increased phagocytosis (Rostalski et al., 2024). Although the different iPSC donors and differentiation protocols used may have influenced the results, there is no simple explanation for this discrepancy. It remains to be shown whether the reported effects truly represent differential kinetics in phagocytic uptake or deficient autophagy-lysosomal degradation due to C9orf72 loss-of-function (Banerjee et al., 2023; Masrori et al., 2025). However, together these data firmly establish impaired microglial function in C9-ALS.

**Gene expression changes:** To determine whether *C9orf72* HRE affects the microglial transcriptome, bulk and single cell RNA sequencing has been used. These studies largely agree that unstimulated C9-ALS microglia only show small transcriptomic changes, in monoculture or co-culture with MNs (Lorenzini et al., 2023; Vahsen et al., 2023; Rostalski et al., 2024; Gao et al., 2026). Nevertheless, alterations in cellular pathways such as metabolism have been identified (Rostalski et al., 2024; Mearelli et al., 2026). However, the differentiation protocol appears to have a major effect on the inflammatory profile, with some changes in pro-inflammatory genes and mediators observed in unstimulated microglia differentiated using a specific protocol (Thiry et al., 2025). These modest transcriptomic changes are likely in part attributable to the absence of neuronal cues, as more pronounced changes are seen in organoids and xenotransplanted cells (as discussed below). Multiple studies have therefore used lipopolysaccharides (LPS) to reveal microglial responses to a pro-inflammatory challenge. Indeed, comparatively high-dose and prolonged LPS priming induced a pro-inflammatory profile in C9-ALS microglia through upregulation of cytokine/chemokine pathways and genes such as *CXCL1* and *CXCL6* (Vahsen et al., 2023). Further, increased expression and release of pro-inflammatory mediators such as interleukin 1 beta (IL-1β), IL-6, and matrix metallopeptidase 9 were found (Banerjee et al., 2023; Vahsen et al., 2023). However, shorter and low-dose priming with LPS resulted in opposite effects (Gao et al., 2026). *C9orf72* knock-out replicated the results after low-dose and high-dose priming, respectively, suggesting these phenotypes are associated with loss-of-function (Banerjee et al., 2023; Gao et al., 2026). Low-dose stimulation could be more reflective of an early phase of the disease, whereas high-dose stimulation might induce late-stage phenotypes. This suggests that C9-ALS microglia may therefore be less or more inflammatory than control cells depending on the context.

**Induction of neuronal toxicity:** Another key readout has been microglial non-cell-autonomous toxicity using conditioned medium and co-culture experiments. These models are more complex, but produce a more realistic microglial identity. There is a relative consensus that C9-ALS microglia reduce the survival of co-cultured MNs. One study reports this without external stimulation (Sonustun et al., 2025), while others required long-term stimulation with microglia-conditioned medium (Thiry et al., 2025), chronic priming with LPS (Vahsen et al., 2023), or excitotoxic treatment with AMPA (Banerjee et al., 2023). This toxicity is, at least partly, mediated by matrix metallopeptidase 9 (Vahsen et al., 2023). Notably, short-term LPS treatment did not trigger microglia-mediated neurotoxicity (Mearelli et al., 2026), in keeping with the view that this may be a more late-stage phenomenon of the disease. It is not yet clear whether this toxicity is associated with loss- or gain-of-function effects of *C9orf72* HRE, or specific to MNs. However, current data argue in favour of an actively toxic microglial phenotype in C9-ALS.

**More complex models:** As outlined above, contrasting changes in microglial gene expression and function have been reported in C9-ALS in different experimental settings. In part, these can be explained by differences in experimental procedures, species or disease stages studied. However, it is becoming clear that microglia are dependent on their three-dimensional (3D) environment and that changes in this environment can have large impact on microglial biology. For example, co-culture with neurons and astrocytes impacts the microglial inflammatory response to LPS, resulting in changes closer to the *in vivo* response. In light of this cellular relationship, several more complex models have been used to assess microglial changes in C9-ALS, including post-mortem tissue, *in vivo* transplantation of microglial progenitors, neural organoids, tri-cultures, and microtissues. Single-nuclei RNA sequencing of human postmortem tissue showed reduced gene expression related to phagocytic and lysosomal pathways in C9-ALS microglia, and an impaired transition into disease-associated microglia and human leukocyte antigen states (Masrori et al., 2025). Interestingly, this reduction was more pronounced in spinal cord as compared to motor cortex, and distinct from changes in sporadic ALS patients (Masrori et al., 2025). These observations were confirmed in a human microglia xenograft mouse model in which *C9orf72* HRE led to reduced activation (Masrori et al., 2025). Interestingly, C9-ALS microglia in another complex tissue model, cerebral organoids, displayed a transcriptomic signature similar to the signatures described in C9-ALS patient material (Ljubikj et al., 2025). Although neural organoids do not represent models of fully matured brain cells, organoid tissue more closely resembles the multicellular environment of the human brain and promotes (non)neuronal maturation (including microglia). Further, oMG show ramified morphology, closely mimic the transcriptome and functional responses of adult microglia acutely isolated from post-mortem human brain tissue, and provide the opportunity of studying microglia in the presence of other cell types. C9-ALS oMGs showed reduced morphological complexity and downregulation of genes involved in phagocytosis, lysosomes and inflammatory response (Ljubikj et al., 2025). In line with this, secretion of inflammatory cues from oMG-containing organoids and LAMP1 expression in oMGs were decreased (Ljubikj et al., 2025). In addition, isolated oMGs performed less phagocytosis and contained less post-synaptic proteins in the organoids (Ljubikj et al., 2025). RNA sequencing identified a PU.1 (SPI1) regulon as the most strongly deregulated transcription factor network in C9-ALS oMGs, and viral-mediated expression of PU.1 in microglial cultures rescued phagocytosis and gene expression deficits (Ljubikj et al., 2025). Other types of multicellular cultures have led to additional insights, such as the role of astrocytes in driving C9-ALS microglia dysfunction and identification of neuroprotective compounds that target microglial neurotoxicity. Tri-cultures of C9-ALS microglia, astrocytes and MNs revealed that microglia-driven metabolic programming in astrocytes contributes to the vulnerability of MNs after pro-inflammatory priming with LPS (Mearelli et al., 2026). Further, a 3D spinal microtissue model for C9-ALS (a scalable approach to combine glial cells and MNs *in vitro*) was used to test U.S. Food and Drug Administration-approved compounds for their potential to target inflammation (Sonustun et al., 2025). Using IL-6 and IL-8 release as an inflammatory readout, this study showed that Telmisartan, an angiotensin II receptor blocker, can reduce inflammation and preserve MN viability in C9-ALS spinal microtissue (Sonustun et al., 2025). Together, these results underscore the need to study microglial changes in multi-cellular disease environments in 3D.

**Future perspectives:** While recent work has firmly established microglial dysfunction in C9-ALS, future studies should exploit models and approaches that capture the cellular, regional, and molecular complexity of the microglial environment. Microglia monocultures have provided crucial insights, but their phenotypes are ultimately determined by interactions with other cells. Therefore, the field should increasingly adopt co-culture, organoid and xenograft approaches that better reflect the 3D disease environment. By assessing additional key microglial pathways and transcriptional regulators across these model systems, such as TREM2/TYROBP, Fc receptors, complement, and MEF2C, it will be possible to build a more comprehensive picture of whether C9-ALS microglia are more or less active. In parallel, the reliance on LPS stimulation has limited our understanding of disease-relevant microglial states. Employing ALS-specific stimuli, such as apoptotic or stressed neurons, pathogenic TDP-43 species, and *C9orf72* HRE-derived DPRs, will enable the discovery of context-dependent responses. A major unresolved question is whether microglial changes in C9-ALS are causative or a consequence of ongoing pathogenesis. Further, whether *C9orf72* HRE drives microglial dysfunction through loss- and/or gain-of-function mechanisms remains largely unknown. Answering these questions will be critical for optimizing antisense oligonucleotide (ASO) therapies. Cell-specific modulation will also be essential for future ASO strategies: In addition to targeting neuronal *C9orf72* HRE-associated pathogenesis, ASO design and dosing regimes should account for microglial dysfunction. ASO therapy should avoid excessive microglial C9orf72 LOF and restore normal microglial C9orf72 expression. Large-scale microglial differentiations using harmonized protocols across many iPSC lines will also be essential to distinguish true patient-specific heterogeneity from technical variation. Potential sex- and age-associated effects in microglial responses remain to be addressed. Additionally, microglial diversity in different CNS regions remains poorly defined: cortical and spinal microglia likely differ in baseline transcriptional states and responses to neuronal stress. Understanding these regional differences in iPSC models and post mortem samples may reveal why specific motor circuits are selectively vulnerable in ALS. Finally, transplantation-based approaches, such as human microglia engraftment into rodent CNS or in human organoid slices, offer powerful opportunities to validate human-specific phenotypes. Collectively, progress will require integration of more physiologically relevant models, systematic comparisons across genetic backgrounds and CNS regions, and mechanistic dissection of mutation-specific effects. These efforts will enable a more precise understanding of microglial contributions to C9-ALS pathogenesis and may ultimately support the development of targeted, cell-type–specific therapeutic strategies.

*BFV is funded by the University of Oxford Medical Sciences Division (Reference 0017772) and the Motor Neurone Disease Association Lady Edith Wolfson Junior Non-Clinical Fellowship (Vahsen/Oct25/2553-799). RJP is funded by Stichting ALS Nederland (TOTALS, ALS-on-a-chip, ATAXALS, MUSALS, GoALS), the ERANet for Research on Rare Diseases (E-rare-3: MAXOMOD and INTEGRALS), the ALS CURE project, Alzheimer Nederland and the EU Joint Program for Neurodegenerative Diseases (JPND; TRIAGE).*
